# The Type III Secretion System (T3SS) is a Determinant for Rice-Endophyte Colonization by Non-Photosynthetic *Bradyrhizobium*

**DOI:** 10.1264/jsme2.ME15080

**Published:** 2015-11-19

**Authors:** Pongdet Piromyou, Pongpan Songwattana, Teerana Greetatorn, Takashi Okubo, Kaori Chiba Kakizaki, Janpen Prakamhang, Panlada Tittabutr, Nantakorn Boonkerd, Neung Teaumroong, Kiwamu Minamisawa

**Affiliations:** 1 School of Biotechnology, Institute of Agricultural Technology, Suranaree University of TechnologyNakhon Ratchasima, 30000Thailand; 2 Department of Applied Biology, Faculty of Sciences and Liberal ArtsRajamangala University of Technology-Isan, 30000Thailand; 3 Graduate School of Life Sciences, Tohoku University2–1–1 Katahira, Aoba-ku, Sendai 980–8577Japan

**Keywords:** Rice (*Oryza sativa*), endophyte, *Bradyrhizobium*, type III secretion system, gene expression, genome

## Abstract

Plant associations by bradyrhizobia have been detected not only in leguminous plants, but also in non-leguminous species including rice. *Bradyrhizobium* sp. SUTN9-2 was isolated from *Aeschynomene americana* L., which is a leguminous weed found in the rice fields of Thailand. This strain promoted the highest total rice (*Oryza sativa* L. cultivar Pathum Thani 1) dry weight among the endophytic bradyrhizobial strains tested, and was, thus, employed for the further characterization of rice-*Bradyrhizobium* interactions. Some known bacterial genes involved in bacteria-plant interactions were selected. The expression of the type III secretion component (*rhcJ*), type IV secretion component (*virD4*), and pectinesterase (*peces*) genes of the bacterium were up-regulated when the rice root exudate was added to the culture. When SUTN9-2 was inoculated into rice seedlings, the *peces*, *rhcJ*, *virD4*, and exopolysaccharide production (*fliP*) genes were strongly expressed in the bacterium 6–24 h after the inoculation. The gene for glutathione-*S*-transferase (*gst*) was slightly expressed 12 h after the inoculation. In order to determine whether type III secretion system (T3SS) is involved in bradyrhizobial infections in rice plants, wild-type SUTN9-2 and T3SS mutant strains were inoculated into the original host plant (*A. americana*) and a rice plant (cultivar Pathum Thani 1). The ability of T3SS mutants to invade rice tissues was weaker than that of the wild-type strain; however, their phenotypes in *A. americana* were not changed by T3SS mutations. These results suggest that T3SS is one of the important determinants modulating rice infection; however, type IV secretion system and *peces* may also be responsible for the early steps of rice infection.

*Aeschynomene americana* is a perennial leguminous weed found in rice fields for only a few months a year (between July and December in Thailand). Bacteria belonging to the genus *Bradyrhizobium* establish symbiotic relationships with this plant, and nitrogen-fixing nodules are formed on the root and/or stems (19). Previous studies indicated that *Bradyrhizobium* species endophytically colonize the roots of certain cereal crop plants, thereby promoting their growth and grain yield at harvest (4, 39, 40). Furthermore, bradyrhizobia are known to directly supply biologically fixed nitrogen to legume plants and also have great potential to improve sustainable rice production (3).

Some endophytic bacteria that reside inside the tissues of healthy plants have the ability to promote biomass production by the host plants (43). However, the potential of applying endophytic bacteria is still underexplored (13, 16). Chaintreuil *et al.* (4) showed that photosynthetic *Bradyrhizobium* (PB) strains from the stem/root nodules of *Aeschynomene* established an endophytic association with the wild rice species *Oryza breviligulata*. Piromyou *et al.* (25) revealed that non-PB (*Bradyrhizobium* sp. SUTN9-2) also had the ability to form a natural endophytic association with cultivated rice plants. Despite the widespread occurrence of this natural endophytic bradyrhizobial-rice association, its colonization and infection processes have not yet been examined in detail.

In order to understand the mechanisms underlying the bradyrhizobial-rice association in more detail, we investigated the gene expression profiles of SUTN9-2 during the early stage of infection in a Thai variety of rice (*O. sativa* L. ssp. *indica* cv. Pathum Thani 1). Previous studies (7, 33) reported that some bacterial genes including exopolysaccharide production (*exoB*), flagella biosynthetic protein (*fliP*), type III secretion component (*rhcJ*), type IV secretion component (*virD4*), pectinesterase (*peces*), and glutathione-*S*-transferase (*gst*) were important for bacteria-plant interactions. In the present study, we constructed mutants of the type III secretion system (T3SS) in *Bradyrhizobium* sp. SUTN9-2, which is strongly expressed in rice plant tissues, and examined their symbiotic phenotypes with rice plants.

## Materials and Methods

### Bacterial strains, plasmids, and growth conditions

The bradyrhizobial strains and plasmids used in this study are listed in Table 1. Bradyrhizobial strains were cultured on HEPES-MES (HM) medium at 28°C (5), and *Escherichia coli* strains were cultured at 37°C in Luria-Bertani medium (29).

### GUS-tagging of endophytic bradyrhizobia and monitoring of root colonization

Bradyrhizobial strains were tagged with mTn5SS*gusA20* (pCAM120) (Table 1) by triparental mating on HM agar plates (5), using pRK2013 as a helper plasmid (38). The cell slurry was mixed with HM and 100 μL of the cell suspension was then plated on HM agar plates containing 100 μg mL^−1^ of streptomycin, 100 μg mL^−1^ of spectinomycin, and 50 μg mL^−1^ of polymycin B. Transconjugant blue colonies were selected in the presence of X-Gluc (40 mL 20 mg mL^−1^ X-Gluc in *N,N*-dimethyl-formamide, 20 mg SDS, 2 mL methanol, 0.2 mL 1 M sodium phosphate buffer, and 7.76 mL distilled water).

Rice (*O. sativa* L. ssp. *indica* cv. Pathum Thani) seeds were surface-sterilized as described previously (25). The seeds were germinated on sterilized wet tissue paper. After 2 d, rice seedlings were transferred into 80-mL tubes containing 10 mL nitrogen-free growth medium (9). One milliliter of GUS-tagged bacteria was inoculated into the growth medium at a density of 10^7^ cells mL^−1^ 3 d after transferring seedlings into the growth medium. Roots were examined for bacterial colonization by GUS staining 7 d after inoculation (dai). In order to detect the expression of β-glucuronidase (GUS) by endophytes in plant tissue, GUS staining was performed as described previously (15). The stained roots were embedded in 5% agarose gel, and 90-μm-thick sections were prepared using a vibratome (Microm HM 650V). Root samples were then directly observed by light microscopy (Carl Zeiss/Primo Star HD microscope [Carl Zeiss^®^, Germany]).

### Scanning electron micrograph (SEM) analysis of *Bradyrhizobium* SUTN9-2 colonization in rice root tissues

Seven days after the inoculation of rice roots with *Bradyrhizobium* SUTN9-2, the tissues were fixed with 2.5% (v/v) glutaraldehyde in 0.1 M sodium phosphate buffer pH 7.2 for 2 h, and then postfixed in 1% (w/v) osmium tetroxide in the same buffer for 2 h. The fixed roots were dehydrated in graded ethanol series. Tissue samples were transferred to a critical point dryer using liquefied carbon dioxide as the transitional fluid. Tissues were mounted on an aluminum cylinder (stub), and finally coated with carbon and gold by vapor deposition (1, 20). Samples were then examined under a scanning electron microscope (JSM 7800F, Japan).

### Preparation of rice root exudates

Rice seeds were surface-sterilized as described above and germinated seeds were then transferred into 80-mL tubes (5 seeds tube^−1^) containing 5 mL nitrogen-free medium (9). In order to obtain rice root exudates, the procedures described by Shidore *et al.* (34) were performed with some modifications. Seven days after transferring seedlings into growth medium, medium solutions containing root exudates were transferred into new tubes. Root exudate solutions were repeatedly sterilized through 0.2-μm membrane filters and then stored at −20°C before being used.

### Rice experiments

Leonard’s jar assembly was filled with sterilized vermiculite, and nutrient solutions were applied through a wick to provide nutrients to plants (9). The whole apparatus was autoclaved (121°C for 25 min) prior to the transplantation of seedlings. Surface-disinfected rice (cultivars Pathum Thani 1 and Nipponbare) seeds were germinated on sterilized filter sheets in Petri dishes. Uniformly germinated seeds were transplanted into Leonard’s jar containing sterilized vermiculite under aseptic conditions (three plants per Leonard’s jar). One milliliter of a 5-d-old inoculum (approximately 10^7^ cells mL^−1^) was inoculated into seedlings 2 d after their transplantation. This experiment was conducted with three replicates per each bacterial strain. Plants were grown under controlled environmental conditions of 28 ± 2°C on a 12-h day/night cycle at a light intensity of 300 μE m^−2^S^−1^ and 50% humidity. Plant dry weights were measured after 30 dai.

### Enumeration of endophytic bradyrhizobia

Roots, leaves, and leaf sheaths were surface-sterilized. As a control to assess the superficial contamination of each individual plant, 20 μL of water from the final rinse was spread on Yeast Mannitol (YM) plates (g liter^−1^; mannitol, 10; K_2_HPO_4_, 0.5; yeast extract, 0.5; NaCl, 0.1; MgSO4·7H_2_O, 0.2). Individual rice samples with bacterial contamination on YM plates were excluded from further endophytic bradyrhizobial enumeration (25). Surface-sterilized roots, leaves, and leaf sheaths were separately macerated with a sterilized mortar and pestle, and then diluted in normal saline solution (0.85% NaCl) prior to spreading on YM plates. In order to enumerate SUTN9-2 Δ*rhcJ* strains, spectinomycin and streptomycin (200 μg mL^−1^ each) were added to YM plates. After a 7-d incubation at 28°C, the number of bacterial colonies was counted to determine bacterial population densities in rice tissues (CFU g^−1^ root fresh weight).

### RNA isolation and reverse transcription PCR amplification

*Bradyrhizobium* sp. SUTN9-2 was grown in HM-modified medium (HM supplemented with 1/3 [vol/vol] of the root exudate solution). Bacterial cells were collected by centrifugation (2,943 × *g* for 10 min) 3 h after root exudate induction. Cell pellets were immediately frozen in liquid nitrogen and stored at −80°C for further total RNA extraction.

Rice seeds were surface-sterilized (25) and germinated on 0.85% water agar for 2 d. Rice seedlings were transferred into 40-mL tubes containing 5 mL nitrogen-free growth medium. Three days after the transferal of seedlings into growth medium, 1 mL of the bacterial culture (10^7^ cells mL^−1^) was inoculated into rice roots. Root samples were collected 6 h, 12 h, 1 d, 3 d, and 7 d after the inoculation. Rice roots were immediately frozen in liquid nitrogen and stored at −80°C for further total RNA extraction. The experiment was laid out with a completely randomized design (CRD) with three biological replications.

Total RNA was extracted from bacterial cells and directly isolated from rice samples using the RNeasy Plant Mini Kit (QIAGEN, USA) according to the manufacturer’s protocol. Following the DNase treatment, RNA samples were resuspended in diethylpyrocarbonate-treated water. Total RNA was treated with 1 U of RNase free DNase (Promega, USA) and incubated at 37°C for 30 min. One microliter of stop solution (20 mM EGTA [pH 8.0] at 25°C) was added and incubated at 70°C for 15 min. RNA was preheated at 70°C for 5 min and reverse transcribed. The reverse transcription reaction mixture was composed of 4 μL of ImProm-II^™^ 5X reaction buffer, 3 mM MgCl_2_, 0.67 mM dNTPs mix, 20 U of ribonuclease inhibitor, and 1 μL of ImProm-II^™^ reverse transcriptase (Promega, USA), and the volume of the solution was adjusted with nuclease-free water to 15 μL. Five microliters of RNA and primer mix were added to the reverse transcription reaction mixture, giving a final reaction volume of 20 μL. The reaction was performed at 25°C for 5 min, extended at 42°C for 60 min, and reverse transcriptase was inactivated at 70°C for 15 min. A reverse-transcription polymerase chain reaction (RT-PCR) was performed using primers designed to target genes (Table 2). Two microliters of cDNA was amplified by PCR under the same conditions as those for DNA amplification. The cycling conditions were an initial denaturation step at 94°C for 3 min; 30 cycles at 94°C for 30 s, 30 s at each annealing temperature (Table 2), and at 72°C for 1.5 min; and a final extension step at 72°C for 10 min. All RT-PCR reactions were performed using a Thermal cycler BIO-RAD T100^™^, and the products were analyzed in 1% agarose gel stained with 0.5 μg mL^−1^ ethidium bromide and visualized in a Gel documentation and analysis system (Gel Doc XR^+^ system, BIO-RAD). 16S rRNA was used as a standard to calibrate the amount of RNA. All RT-PCRs were performed in triplicate on cDNA from three independent biological replicates.

### Genome analysis and comparison

Cells of *Bradyrhizobium* sp. SUTN9-2 were cultured at 30°C in HM salt medium (5). In the stationary phase, cells were harvested by centrifugation, and total DNA was prepared as previously described (17). The 8-kb paired-end 454 library of SUTN9-2 was constructed from total DNA by Takara Bio (Shiga, Japan). Genome sequencing was performed using a 454 GS FLX+ sequencer (Roche Diagnostics K.K.; Tokyo, Japan). Generated sequences were assembled using Roche GS De Novo Assembler version 2.8 (Roche Diagnostics K.K.; Tokyo, Japan). Predictions of gene regions and annotation were performed using MiGAP (http://www.migap.org/index.php/en/about-migap). A circular genome map showing the GC skew and GC content was created using the GCview Server (http://stothard.afns.ualberta.ca/cgview_server).

The genome sequences of *Bradyrhizobium* sp. SUTN9-2 (accession number LAXE00000000) were compared with the whole genome of *B. diazoefficiens* USDA110 (33) using the program GenomeMatcher (21) at the amino acid level. The annotated genome sequences of USDA110 (accession number BA000040) were obtained from Genome Assembly/Annotation Projects (NCBI database). The sequences of chromosome and plasmid pDOA9 are available in the DDBJ/GenBank/EMBL database (accession numbers DF820425 and DF820426, respectively).

### Construction of the *rhcJ* mutant

In order to construct *Bradyrhizobium* sp. SUTN9-2 deletion mutants (*rhcJ*), DNA fragments corresponding to the upstream and downstream regions of the *rhcJ* gene obtained from SUTN9-2 draft genome data (Fig. S3) were individually amplified by PCR (Table 2). DNA amplification from the upstream fragment (1,065-bp *Kpn*I-*Bam*HI) and downstream fragment (1,059-bp *Bam*HI-*Xba*I) were obtained from the genomic DNA of SUTN9-2. The upstream fragment (1,065-bp *Kpn*I-*Bam*HI) and downstream fragment (1,059-bp *Bam*HI-*Xba*I) were ligated into the pGEM^®^-T Easy vector (Promega), yielding the plasmids pGEM^®^-TUP and pGEM^®^-TDOWN, respectively. pGEM^®^-TUP and pGEM^®^-TDOWN were digested using the restriction enzymes *Kpn*I and *Bam*HI. The upstream fragment (1,065-bp *Kpn*I-*Bam*HI) from pGEM^®^-TUP was then cloned into pGEM^®^-TDOWN, generating the plasmid pGEM^®^-TUP/DOWN. pGEM^®^-TUP/DOWN was digested using *Kpn*I and *Xba*I and the digested fragment was subcloned in the *Kpn*I and *Xba*I sites of the plasmid pK18mobsacB (31) to yield the plasmid pK18mobsacB-UP/DOWN. The antibiotic gene (spectinomycin/streptomycin) Omega cassette (27) was inserted into the *Bam*HI site of pK18mobsacB-UP/DOWN (between the upstream and downstream fragments) to yield the plasmid pK18mobsacB-UP/DOWN/Spc_r_/Sm_r_. The plasmid pK18mobsacB-UP/DOWN/Spc_r_/Sm_r_ was mobilized into *Bradyrhizobium* sp. SUTN9-2 through triparental mating with the helper plasmid pRK2013 (38). The *rhcJ* gene was deleted by homologous recombination in *Bradyrhizobium* sp. SUTN9-2, generating the mutant SUTN9-2 Δ*rhcJ* (Table 1). Marker exchange was forced by selection on HM agar plates containing 10% (wt/vol) sucrose. The events of double crossover around the *rhcJ* gene were verified by PCR (Fig. S1).

### Plant nodulation tests and nitrogen fixation assay

Bradyrhizobial strains were grown for 5 d in HM broth. All plants were grown in a growth chamber with controlled environmental conditions of 28 ± 2°C on a 12-h day/night cycle at a light intensity of 300 μE m^−2^S^−1^ and 50% humidity. Mung bean (*Vigna radiata*) and siratro (*Macroptilium atropurpureum*) seeds were sterilized (35). The seeds of *A. americana* (a local Thai variety) were sterilized by an incubation in concentrated sulfuric acid for 25 min (19). The nodulation test was performed in sterilized plastic pouches with five replicates. The root nodules from the tested plants were enumerated at 30 dai.

The symbiotic abilities of bradyrhizobial strains were determined in Leonard’s jars containing sterilized vermiculite, and 1 mL of each bacterial strain equivalent to 10^7^ cells was inoculated onto germinated *A. americana*, *V. radiata*, and *M. atropurpureum* seeds. Plants were harvested after 30 d, and entire plants were used to analyze nitrogenase activity using the acetylene reduction assay (ARA) (26).

### Statistical analyses

Experimental data were statistically analyzed according to Steel and Torrie (36), and means were compared by Duncan’s Multiple Range Test (8).

## Results

### Localization of rice endophytic bradyrhizobial strains

**Fig. 1:**
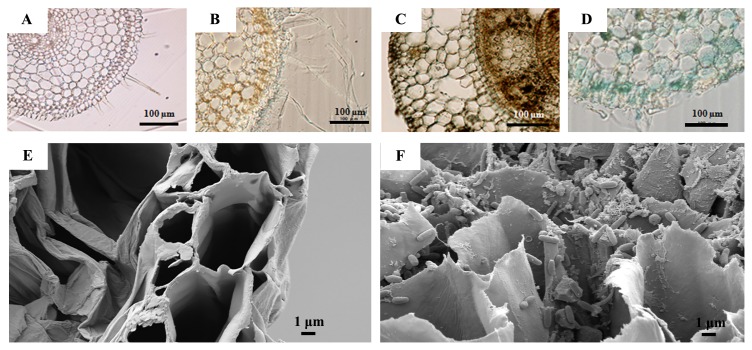
Localization of bradyrhizobial strains inside rice root tissues based on light and scanning electron microscopic observations. Bacterial localization in rice roots 7 d after inoculation (dai) by GUS-tagged USDA110 (A), DOA1 (B), DOA9 (C), and SUTN9-2 (D). Scanning electron micrograph (SEM) of rice roots without bacterial inoculation (E) and with SUTN9-2 at 7 d (F).

Since many bradyrhizobial strains have been isolated from the nodules of *A. americana*, we herein selected five representative strains (SUTN8-1, SUTN9-2, DOA1, DOA4, and DOA9) based on five clusters of the 16S rRNA phylogenetic tree (19) (Fig. S2). In an attempt to determine whether these selected strains were rice endophytes, they were tagged with the GUS reporter gene, and their localization was observed under a light microscope (Fig. 1). *Bradyrhizobium* sp. strains SUTN9-2, DOA1, and DOA9 were clearly identified inside the rice tissues (cultivar Pathum Thani 1) at 7 dai based on GUS reporter gene detection. Strain SUTN9-2 colonized well in the intercellular spaces of the rice root tissues (Fig. 1D). SEM was also used to confirm the localization of SUTN9-2 inside rice root tissues (Fig. 1E and 1F), and the results obtained were consistent with those from the GUS-tagging experiment.

### Total plant dry weight and colonization

**Fig. 2:**
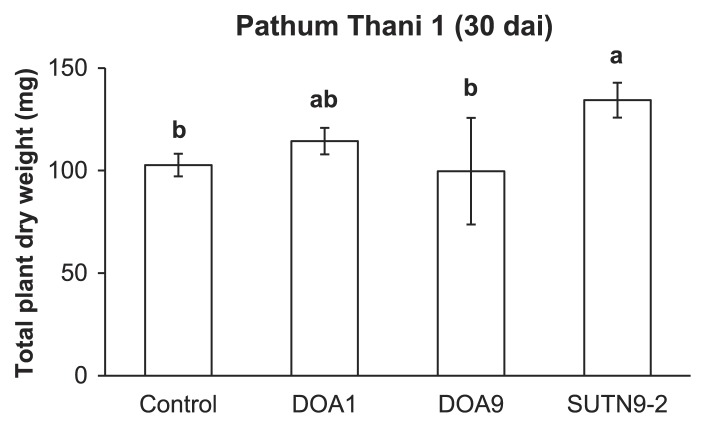
Effects of endophytic *Bradyrhizobium* on the biomass of rice cultivars (*O. sativa* L. ssp. *indica* cv. Pathum Thani 1). Significance at *P* < 0.05 is indicated by mean standard deviation bars (*n* = 3).

Prior to investigating the effects of bradyrhizobial strains on rice biomass, the nitrogen source for plants at 0, 0.1, and 1 mM of NH_4_NO_3_ was applied to N-free medium. Of the three concentrations of NH_4_NO_3_ tested, 0.1 mM NH_4_NO_3_ caused a significant difference in total plant dry weights between bradyrhizobial inoculation treatments and the uninoculated control (data not shown). Therefore, 0.1 mM NH_4_NO_3_ was subsequently used to determine the total plant dry weight at 30 dai. The total dry weight of plants inoculated with SUTN9-2 was significantly higher than that of the uninoculated control (Fig. 2), whereas no significant difference was observed between the DOA1 and DOA9 inoculations and the uninoculated control (Fig. 2).

**Fig. 3:**
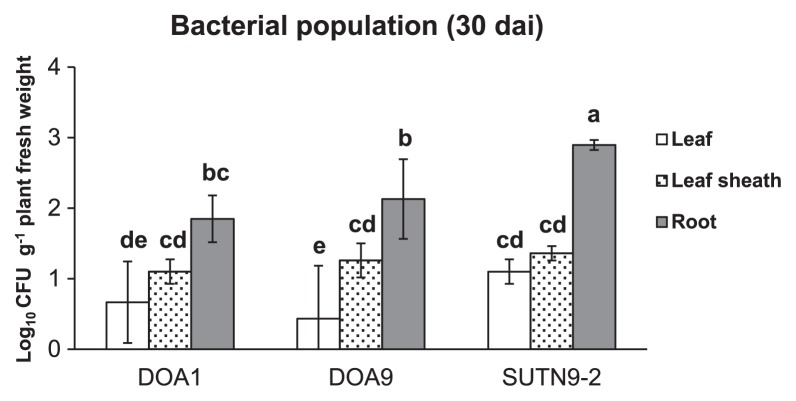
Enumeration of bradyrhizobia in the leaf, leaf sheath, and root of rice tissues. The populations of endophytic bradyrhizobia were examined in the tissues of *O. sativa* L. ssp. *indica* cv. Pathum Thani 1 at 30 d after inoculation (dai). Significance at *P* < 0.05 is indicated by mean standard deviation bars (*n* = 3).

The bradyrhizobial population (in the range of 2–3 log_10_CFU g^−1^ tissue fresh weight) in rice (cultivar Pathum Thani 1) root tissues at 30 dai was larger than that in the leaf sheath and leaf (Fig. 3). The SUTN9-2 population inside root tissues was significantly larger than those of strains DOA1 and DOA9. However, no significant differences were observed in the bacterial population inside the leaf sheath between any of the treatments used.

Since SUTN9-2 had the highest cell density in rice tissues and rice growth promotion, it was selected for subsequent examinations on the mechanisms underlying the rice-bradyrhizobia association.

### SUTN9-2 gene expression analysis of the rice association

**Fig. 4:**
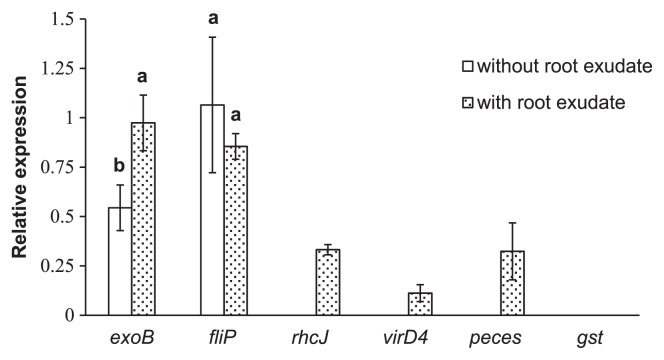
Relative expression levels of selected genes of *Bradyrhizobium* sp. SUTN9-2 incubated with and without rice root exudate induction. The letters inside parentheses show the target genes; exopolysaccharide production (*exoB*), flagella biosynthetic protein (*fliP*), type III secretion component (*rhcJ*), type IV secretion component (*virD4*), pectinesterase (*peces*), and glutathione-*S*-transferase (*gst*). The 16S rRNA gene was used as an internal control. Significance at P ≤ 0.05 is indicated by mean standard deviation bars (*n* = 3).

In order to detect the expression of genes (Table 2) relevant to the rice association at the early stage in SUTN9-2, rice root exudates were supplemented into bacterial cultures. The up-regulated expression of the *exoB* gene was significantly greater in cultures supplemented with rice root exudate than in those without exudate (Fig. 4). On the other hand, no significant differences were observed in the relative expression levels of the *fliP* gene between cultures supplemented with and without root exudate. The *rhcJ*, *virD4*, and *peces* genes were only expressed when rice roots exudates were added, whereas the expression of *gst* was not detected 3 h after root exudate induction. The *rhcJ*, *virD4*, *peces*, and *gst* genes were not expressed with the uninduction treatment (Fig. 4).

**Fig. 5:**
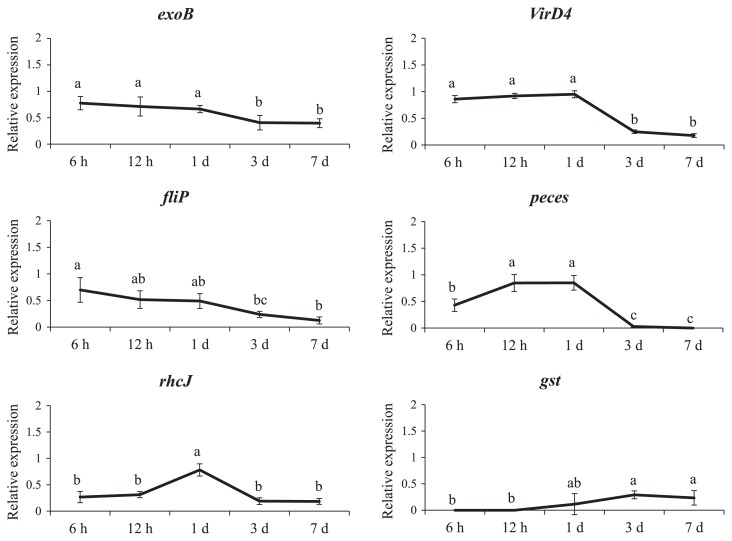
Relative gene expression of selected genes in *Bradyrhizobium* sp. SUTN9-2 in rice plants (*in planta*). The letters inside parentheses showed the target genes; exopolysaccharide production (*exoB*), flagella biosynthetic protein (*fliP*), type III secretion component (*rhcJ*), type IV secretion component (*virD4*), pectinesterase (*peces*), and glutathione-*S*-transferase (*gst*). 16S rRNA genes were used as an internal control. Significance at *P* ≤ 0.05 is indicated by mean standard deviation bars (*n* = 3).

*In planta* (endophytic bacterial RNA isolation from rice samples), several genes such as *exoB*, *rhcJ*, *virD4*, and *peces* were strongly expressed 6–24 h after the inoculation (Fig. 5). Furthermore, the *gst* gene was slightly expressed 12 h after the inoculation. On the other hand, the expression of *fliP* slightly decreased with increases in the plant age.

### Comparative analysis of gene clusters

Draft genome sequences were determined in the present study to investigate the organization and functions of genes in SUTN9-2 (Fig. S3). Based on the results of the gene expression experiment (Figs. 4 and 5), gene clusters of *fliP*, *exoB*, T3SS, and T4SS were subjected to gene organization analyses. The flagella formation genes of strain SUTN9-2 were similar to those of *B. diazoefficiens* USDA110 in terms of gene sequences and orientation (Fig. S4). Most of the flagella genes were compactly clustered in two regions including clusters 1 and 2. Since these gene clusters control the flagella formation, their destruction may cease bacterial responses to plants. Moreover, the genes for exopolysaccharide production of strain SUTN9-2 were highly conserved among the strains compared (Fig. S5). Thus, *fliP* and *exoB* genes were not selected for the gene disruption experiment.

**Fig. 6:**
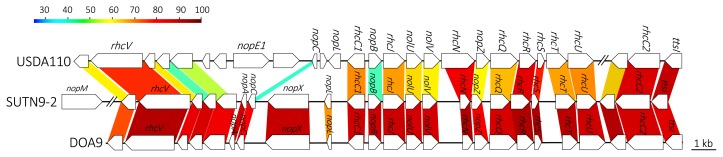
Comparison of the gene organization of the type III secretion system (T3SS) in strains USDA110, SUTN9-2, and DOA9. Double slash marks represent DNA regions that are not shown. Colored strips represent the conserved gene regions between the compared strains, and the color indicates the percentage similarity.

Amino acid sequence comparisons of the T3SS apparatus among non-endophytic *B. diazoefficiens* USDA110 and rice endophytic strains SUTN9-2 and DOA9 revealed low conserved gene sequence and organization (Fig. 6). The amino acid sequences of T3SS of SUTN9-2 showed high similarity with those of DOA9. The ORF of the putative effector gene (*nopE1*) was not detected around T3SS gene clusters in rice endophytic strains SUTN9-2 and DOA9. The putative *nopA* and *nopX* were only present in the T3SS cluster of rice endophytic strains SUTN9-2 and DOA9. The putative *nopM* was only detected in the T3SS cluster of SUTN9-2. These results were interesting because the T3SS of SUTN9-2 was similar to rice endophytic strain DOA9. Therefore, T3SS was selected for the gene deletion experiment.

**Fig. 7:**
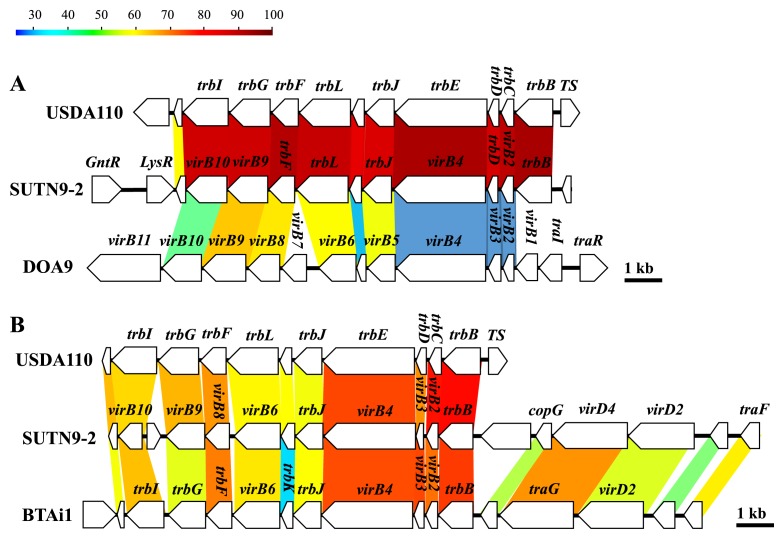
Comparison of the gene organization of the type IV secretion system (T4SS) in strains USDA110, SUTN9-2, and DOA9. Panels A and B were presented as T4SS cluster 1 and T4SS cluster 2, respectively. Colored strips represent the conserved gene regions between the compared strains, and the color indicates the percentage similarity.

The type IV secretion system (T4SS) of SUTN9-2 was found to be encoded in two clusters (Fig. 7). T4SS cluster 1 (Fig. 7A) in *B. diazoefficiens* USDA110 and SUTN9-2 showed high similarities in their amino acid sequences and gene organization, except for the direction of the transcriptional regulator (*LysR*), which was different. In contrast, the T4SS cluster arrangement of DOA9 partially differed from that of SUTN9-2. The amino acid sequences of DOA9 shared approximately 30–63% similarity with those of strains SUTN9-2 and USDA110. The gene arrangement in T4SS cluster 2 of SUTN9-2 was highly similar to T4SS of *Bradyrhizobium* sp. BTAi1 (Fig. 7B). T4SS was not considered for the gene disruption experiment because the T4SS organization and amino acid sequences of SUTN9-2 were markedly different from those of rice endophytic strain DOA9.

### Nodule formation and rice-bradyrhizobia association by T3SS mutants

**Fig. 8:**
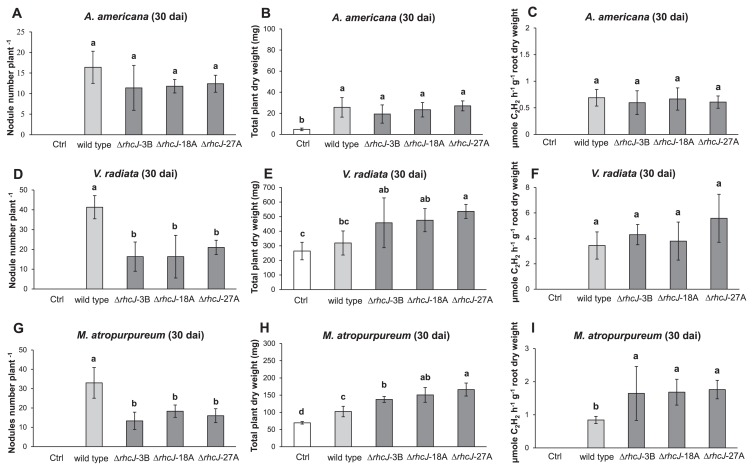
Nodulation and plant growth promotion by leguminous plants inoculated with wild-type SUTN9-2 and three Δ*rhcJ* mutant strains. Nodule numbers (A, D, and G), total dry weights (B, E, and H), and ARA (C, F, and I) are shown for three different legumes (ABC, *A. americana*; DEF, Mung bean; GHI, Siratro). Significance at *P* < 0.05 is indicated by mean standard deviation bars (*n* = 5).

**Fig. 9:**
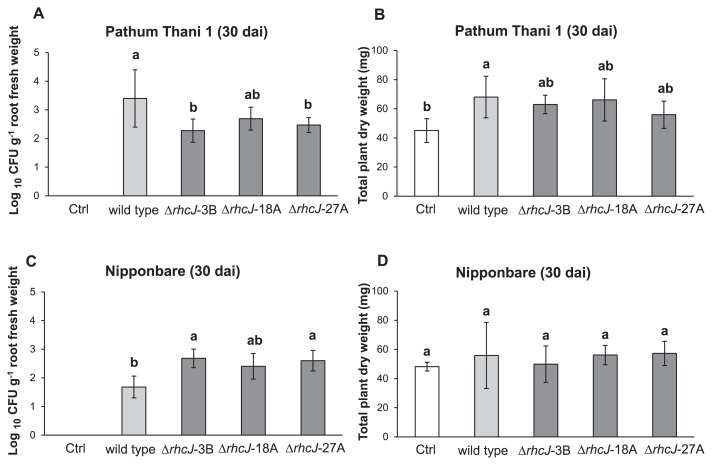
Rice colonization and growth promotion by SUTN9-2 wild-type and T3SS mutant strains (Δ*rhcJ*-3B, Δ*rhcJ*-18A, and Δ*rhcJ*-27A) with *O. sativa* cultivar Pathum Thani 1 (A and B represent the bacterial population and total plant dry weight, respectively) and *O. sativa* cultivar Nipponbare (C and D represent the bacterial population and total plant dry weight, respectively) at 30 dai. Significance at *P* < 0.05 is indicated by mean standard deviation bars (*n* = 3).

The symbiotic characteristics of wild-type SUTN9-2 and T3SS mutants (Δ*rhcJ*-3B, Δ*rhcJ*-18A, and Δ*rhcJ*-27A) were examined using the plant hosts *A. americana* (Fig. 8) and rice cultivar Pathum Thani 1 (Fig. 9). In the original host plant (*A. americana*) of SUTN9-2, the nodule numbers formed by the T3SS mutants were not significantly different from those of wild-type strain SUTN9-2 (Fig. 8A). Furthermore, the plant dry weights and nitrogenase activities of nodules derived from wild-type and *rhcJ* mutant strains were not significantly different (Fig. 8B and 8C). A previous study (19) suggested that SUTN9-2 was isolated from the root nodules of *A. americana* and had the ability to nodulate a wide range of leguminous plants including *Macroptilium atropurpureum* (siratro), *Arachis hypogaea*, and *Vigna radiata* (mung bean). Therefore, the symbiotic phenotypes of wild-type SUTN9-2 and *rhcJ* mutant strains were also investigated with two leguminous plants including *V. radiata* and *M. atropurpureum*. Wild-type SUTN9-2 formed a significantly larger number of root nodules with *V. radiata* L. cv. SUT4 than *rhcJ* mutants (Fig. 8D). On the other hand, *V. radiata* (mung bean) inoculated with *rhcJ* mutants appeared to promote higher total plant dry weights than that inoculated with the wild-type (Fig. 8E). No significant differences were observed in the nitrogen fixation activity of *rhcJ* mutant strains and the wild-type strain (Fig. 8F). However, many reddish nodules (approximately 10 to 15 nodules plant^−1^) were detected in mung bean-inoculated mutants (data not shown). *M. atropurpureum* (siratro) showed similar results to *V. radiata*; the mutants established a smaller number of symbiotic root nodules than the wild-type strain (Fig. 8G). In contrast, growth promotion and nitrogen fixation by *M. atropurpureum* were significantly higher when derived from mutant strains than from the wild-type strain (Fig. 8H and 8I).

When the wild-type SUTN9-2 and T3SS mutants were inoculated into the rice cultivar Pathum Thani 1, the cell numbers of mutant strains from rice root tissues were significantly lower than that of the wild-type strain (approximately 1 magnitude), except for strain Δ*rhcJ*-18A (Fig. 9A and S6). In contrast, the proliferation of the bacterial wild-type strain inside the leaf sheath was not significantly different from that of mutant strains (Fig. S6). The wild-type and mutant strains did not significantly contribute to the plant biomass. However, the ability of the wild-type strain was able to promote total rice dry weight (cultivar Pathum Thani 1) than that of the uninoculated control. The *rhcJ* mutant strains enhanced the rice biomass more than the uninoculated control (Fig. 9B). In contrast, the population of mutants was significantly higher than that of the wild-type strain when they were tested with *O. sativa* L. ssp. *japonica* cv. Nipponbare (Fig. 9C). In the case of the rice cultivar Nipponbare, rice growth promotion was not detected when the cultivar was inoculated with the wild-type and *rhcJ* mutant strains (Fig. 9D). These results suggested that T3SS positively and negatively affected the colonization of SUTN9-2 in these rice plants, and reduced the growth of the rice cultivar Pathum Thani 1.

## Discussion

Rice root exudates may be one of the factors inducing a higher chemotactic response for endophytic bacteria than the other beneficial soil bacteria present in the rice rhizosphere (2). Although *Bradyrhizobium* species are known as endosymbionts in the root nodules of legume plants such as soybeans, bradyrhizobia also play roles in endophytes that colonize non-leguminous plant tissues and promote plant growth (4, 39). However, the symbiotic interaction between endophytic bradyrhizobia and rice has not yet been elucidated in detail. The present study demonstrated that the *rhcJ* (T3SS), *virD4* (T4SS), and *peces* genes of *Bradyrhizobium* sp. SUTN9-2 are transiently expressed following exposure to the rice root exudate (Figs. 4 and 5), suggesting that their expression is crucial for the association with rice plants. The T3SS mutants of SUTN9-2 indicated that T3SS modulated the level of endophytic colonization, which depended on rice cultivars (Fig. 9).

The outcome of plant-bacterium interactions is often emphasized by bacterial protein secretion systems. T3SS is commonly used by symbiotic and/or pathogenic bacteria to secrete effecter proteins into host cells and, thus, trigger host cell responses (7). The draft genome data displayed a lower G+C content in the T3SS region (58.3%) than that of the chromosome (63.89%). SUTN9-2 may have acquired T3SS through horizontal gene transfer, which often occurs in rhizobia (28). Our results demonstrated that the T3SS gene (*rhcJ*) was strongly expressed when only roots exudates were applied. In addition, the genetic organization and amino acid sequences of T3SS genes were conserved better in both of the rice endophytic strains (SUTN9-2 and DOA9) than in the non-rice endophytic strain USDA110 (Fig. 6). The gene cluster of T3SS components is normally found in the genomes of various bradyrhizobia including non-photosynthetic strains (*B. japonicum* USDA6, *B. diazoefficiens* USDA110, and *B. elkanii* USDA61) and PB strains (*Bradyrhizobium* sp. ORS285 and *B. oligotrophicum* S58). Although the T3SS structural genes of SUTN9-2 (*rhc* and *ttsI* genes) are conserved well with the non-PB group, the putative effector proteins (product of *nop* genes) inside the T3SS cluster were more diverse than those in USDA110 (Fig. 6). These results suggest that T3SS-dependent variable effectors are involved in plant associations; however, the T3SS apparatus appears to have the same evolutionary origin as other bradyrhizobia. These findings implied that the T3SS-dependent system of SUTN9-2 has evolved to respond to various host plants.

The population of SUTN 9-2 was larger in the Thai rice cultivar Pathum Thani 1 than in japonica rice Nipponbare (tentatively by 1 magnitude) (Fig. 9). This result was consistent with previous findings showing that the rice cultivar is one of the factors controlling the compatibility of the rice-bacteria association (25, 30). Although the cell densities of *rhcJ* mutant strains (Δ*rhcJ*) from rice (Pathum Thani 1) tissues were also significantly lower than that of the wild-type strain, this phenomenon was not detected in japonica rice (Nipponbare). These results implied that rice developed a system to protect itself from non-native soil bacteria. In the case of the rice cultivar Pathum Thani 1, SUTN 9-2 had the ability to partially overcome plant defense responses through the function of T3SS; however, this property was not detected in Nipponbare. Therefore, these results may contribute to the confirmation of bradyrhizobia-host evolution.

The Δ*rhcJ* mutant strains established fewer root nodules (*V. radiata* L. cv. SUT4 and *M. atropurpureum*) than the wild-type strain, whereas the number of reddish nodules and nitrogenase activity derived by Δ*rhcJ* mutant strains were greater than those of the wild-type strain. These results were consistent with previous findings reported by Tsukui *et al.* (41), in which the effectors secreted via T3SS triggered incompatibility between plant-bacterial partners. Thus, *V. radiata* L. cv. SUT4 and *M. atropurpureum* may have evolved incompatibility with *Bradyrhizobium* sp. SUTN9-2 in order to prevent their inferior symbionts by using pathogenic systems (Figs. 8 and 9). *B. elkanii* also used T3SS to activate host symbiosis signaling, thereby promoting its infection (22). These results demonstrated that not only nod factors, but also T3SS were important for plant-bradyrhizobia interactions. This is the first study to report that the non-PB strain SUTN9-2 may also use T3SS to form an endophytic association with rice and leguminous symbiosis.

Previous studies revealed that some cultivated rice endophytic bacteria such as *Burkholderia phytofirmans* PsJN and *Herbaspirillum seropedicae* strain SmR1 also contain T3SS genes in their genomes (18, 24). In addition, the proteins secreted by rhizobia have homologues in pathogenic bacteria and may have been co-opted by rhizobia for symbiotic purposes (6). However, the identities of the transcriptional activators of pathogenic bacteria or rice endophytic bacteria currently remain unclear. SUTN9-2 may share some mechanisms with pathogenic or endophytic bacteria in order to respond to signal molecules (such as low molecular weight C-sources and amino acids) in rice root exudates.

The amino acid sequences and organization of the T4SS genes (cluster 1) of SUTN9-2 showed high similarities with those of USDA110 (Fig. 7A). On the other hand, they were less conserved than those of DOA9. The *trp* operon was essential for the conjugative transfer of the Ti-plasmid in *Agrobacterium tumefaciens*. T4SS (cluster 1) appears to play a role in the transferal of genetic elements (14). In T4SS (cluster 2), SUTN9-2 possesses *VirB*/*D4* genes that are similar to *Bradyrhizobium* sp. BTAi1, but are absent in USDA110 (Fig. 7B). The *vir* cluster included the genes *virB2* to *virB10*, the products of which form the transmembrane complex and pili required for the transferal of proteins, while *virA* and *virG* are transcriptional regulator genes. However, the *virB/D4* of T4SS has never been detected in bradyrhizobia, except for *Bradyrhizobium* sp. BTAi1 (23). Moreover, transcriptional regulator genes (*virA* and *virG* genes) were not detected in either of the genomes of *Bradyrhizobium* spp. SUTN9-2 and BTAi1 (data not shown). Nevertheless, *virA* and *virG* homologues were found in the *Mesorhizobium loti* R7A genome (11, 12, 37). These findings suggest that the T4SS (cluster 2) of SUTN9-2 and BTAi1 share the same origin, but also raise another hypothesis that SUTN9-2 acquires the *vir* operon from the PB strain as BTAi1. Therefore, SUTN9-2 may be an intermediate between PB and non-PB evolution. T4SS may be important for *Bradyrhizobium*-rice interactions because *Bradyrhizobium* sp. BTAi1 is also a wild rice endophyte (4).

The *gst* gene encoding the GST of SUTN9-2 appeared to be induced 3 d after the bacterial inoculation (Fig. 5). Plants produce a range of reactive oxygen species (ROS) in response to abiotic stress or colonizing microorganisms. Bacterial GST is generally involved in the detoxification of ROS and products of oxidative stress (10). The genes encoding GST (26 copies), SDS (2 copies), and peroxidases (3 copies), which are involved in the scavenging of ROS, were detected in SUTN9-2. This is supported by previous findings from bacterial genomes. Therefore, numerous genes that encode enzymes for the detoxification of ROS, such as catalase, superoxide dismutase, peroxidases, hydroperoxide reductase, and GST, were detected in various species of bacterial endophytes. A markedly larger number of GST genes were found in the SUTN9-2 genome than in the broad host range endophytic bacteria, *Burkholderia phytofirmans* PsJN, which had 24 copies (18). GSTs are enzymes that detoxify endobiotic and xenobiotic compounds by covalently linking glutathione to hydrophobic substrates and degrading secondary metabolites (18, 42). Based on these functions of GSTs, we speculate that the large varieties of detoxification enzymes are related to the broad host range of SUTN9-2.

Plant-polymer-degrading enzymes are normally present in the bacterial genome and may contribute to endophyte entry and spread inside rice root tissues (33). SUTN9-2 *peces* was only expressed with rice root exudate induction. This enzyme may be one of the factors affecting the rice-*Bradyrhizobium* association.

## Conclusion

The results of the present study demonstrated that bradyrhizobial strains established interactions with not only leguminous plants, but also non-leguminous plants such as rice. The T3SS, T4SS, and *peces* of SUTN9-2 may play important roles in the early stage of rice infection. This study also identified T3SS as one of the important determinants of *Bradyrhizobium* in the endophytic colonization of rice.

## Supplementary Material



## Figures and Tables

**Table 1:** Strains and plasmids used in this study, sampling sites, and relevant characteristics.

Strain or Plasmid	Relevant characteristics and source of isolation	Source or reference
**Strains**		
*Bradyrhizobium* sp.		
DOA1	*A. americana* nodule isolate (paddy crop)	(19)
DOA9	*A. americana* nodule isolate (paddy crop)	(19)
SUTN9-2	*A. americana* nodule isolate (paddy crop)	(19)
DOA1GUS	DOA1 marked with mTn*5*SS*gusA20* (pCAM120); Sm^r^ Sp^r^	This study
DOA9GUS	DOA9 marked with mTn*5*SS*gusA20* (pCAM120); Sm^r^ Sp^r^	This study
SUTN9-2GUS	SUTN9-2 marked with mTn*5*SS*gusA20* (pCAM120); Sm^r^ Sp^r^	This study
Δ*rhcJ*-3B	SUTN9-2 derivative, *rhcJ*::Ω cassette; Sp^r^, Sm^r^	This study
Δ*rhcJ*-18A	SUTN9-2 derivative, *rhcJ*::Ω cassette; Sp^r^, Sm^r^	This study
Δ*rhcJ*-27A	SUTN9-2 derivative, *rhcJ*::Ω cassette; Sp^r^, Sm^r^	This study
*Escherichia coli* S17-1	*pro recA* RP4-2(Tc^s^::Mu) (Km^s^::Tn*7*); Mob^+^	
*E. coli* DH5α	*recA*; cloning strain	Toyobo Inc. (Tokyo, Japan)
**Plasmids**		
pRK404	Broad-host-range vector	(32)
pCAM120	mTn*5*SS*gusA20* in pUT/mini-Tn*5*	Shin Okazaki^a^
pHP45Ω	Plasmid carrying a 2.0-kb Ω cassette; Sp^r^, Sm^r^, Ap^r^	(27)
pK18mob-sacB	Cloning vector; pMB1ori oriT, oriV, sacB; Km^r^	(31)
pRK2013	ColE1 replicon carrying RK2 transfer genes; Km^r^	(38)
pK18mobsacB-UP/DOWN/Spc^r^/Sm^r^	pK18mobsacB carrying 2.123-kb *rhcJ* deletion fragment; Km^r^, Spc^r^, Sm^r^	This study

a Department of International Environmental and Agricultural Science, Graduate School of Agriculture, Tokyo University of Agriculture and Technology, Tokyo, Japan.

**Table 2:** Primers used in this study.

Target genes	Primer names	Primer sequence (5′→3′)	Annealing temperature used (°C)
16S rRNA	fD1	AGAGTTTGATCCTGGCTCAG	45
	rP2	ACGGCTACCTTGTTACGACTT	
*exoB*	exoBF	GAATGAGGTGGGTTTCGTTG	55
	exoBR	GCCCGTCGTTTATGACAATC	
*fliP*	fliPF	GATCAGGAACGGCAGGAATA	50
	fliPR	GGACATCAGCATCAATCTCG	
*rhcJ*	rhcJF	CCCGCTACGTCTATGCTCT	50
	rhcJR	TATTTCGGATCGGAGGACAG	
*Pectin-esterase*	pecesF	TATCACTCCGTGCAACAAGC	49
	pecesR	CGGCACTTAGAGTGCAATGA	
*virD4*	virD4F	CGTCAAGCATCATCAGCACT	55
	virD4R	GAACCATTGGGAGAAGACCA	
*gst*	GST_F	GACCTGAAGCTGATCGAGGA	53
	GST_R	AGATAGTCGATCGCCGAAAG	
Upstream fragment of the *rhcJ* gene	RhcJ-UF	GGGGTACCCCCTTGAACGCATCAAAGCTGA	65
	RhcJ9_2UR	CGGGATCCCGGGGATGAACTGACAGCACCT	
Downstream fragment of the *rhcJ* gene	RhcJ-DF	CGGGATCCCGTGTCCTCCGATCCGAAATAG	62
	RhcJ-DR	GCTCTAGAGCCATACTTCTGCGCTGCCATA	
