# Supplementary material for: The Type III Secretion System (T3SS) is a Determinant for Rice-Endophyte Colonization by Non-Photosynthetic Bradyrhizobium
Source: Microbes Environ. 2015 Nov 19;30(4):291–300. doi: 10.1264/jsme2.ME15080 (PMC4676552; doi:10.1264/jsme2.ME15080)
Supplement: Supplementary file 1 [file 30_291_s1.pdf]

**Supplementary matterials**

# The Type III secretion system (T3SS) is one of determinants for rice-endophyte colonization by non-photosynthetic Bradyrhizobium

Pongdet Piromyou[1], Pongpan Songwattana[1], Teerana Greetatorn[1], Takashi Okubo[3], Kaori Chiba Kakizaki [3], Janpen Prakamhang[2], Panlada Tittabutr[1], Nantakorn Boonkerd[1], Neung Teaumroong[1*], Kiwamu Minamisawa[3*]

School of Biotechnology, Institute of Agricultural Technology, Suranaree University of Technology, Nakhon Ratchasima, 30000, Thailand[1]

Department of Applied Biology, Faculty of Sciences and Liberal Arts, Rajamangala University of Technology-Isan, 30000, Thailand[2]

Graduate School of Life Sciences, Tohoku University, 2-1-1 Katahira, Aoba-ku, Sendai 980-8577, Japan[3]

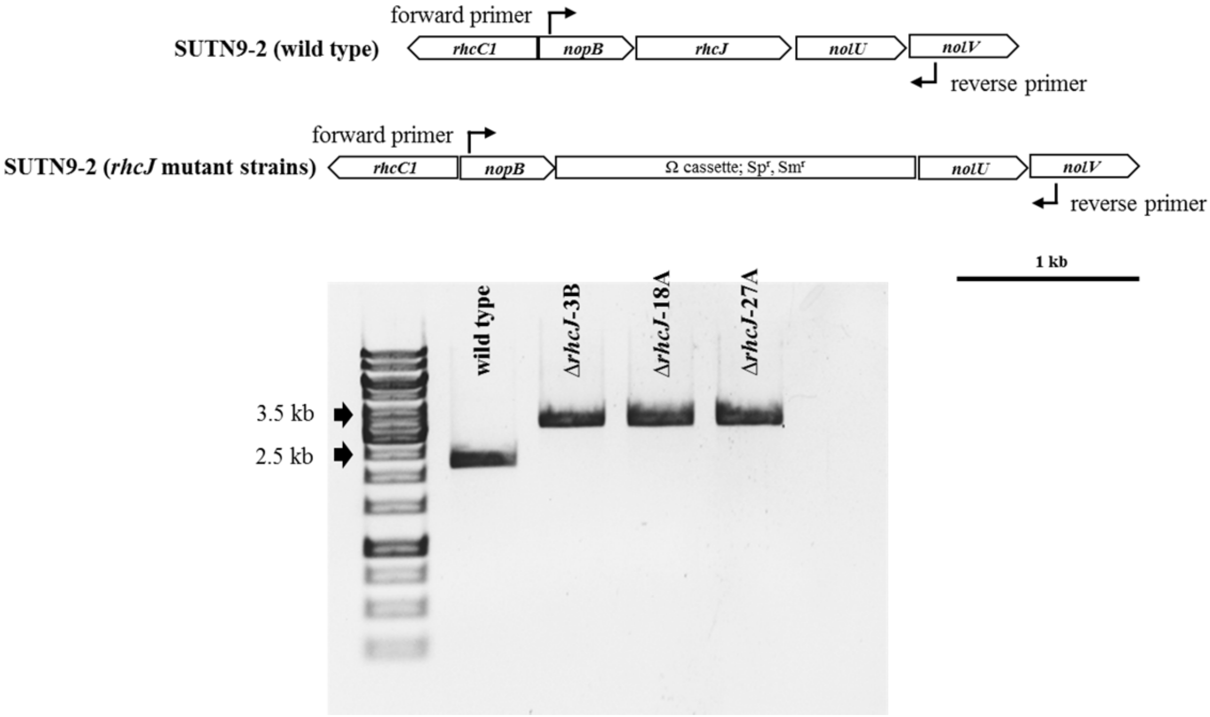


**Fig. S1.** PCR verification of *rhcJ* mutants by using forward primer (5’-cgtcccaaattggaagctaa-3’) and reverse primer (5’-acatctcacccggatcaaag-3’).

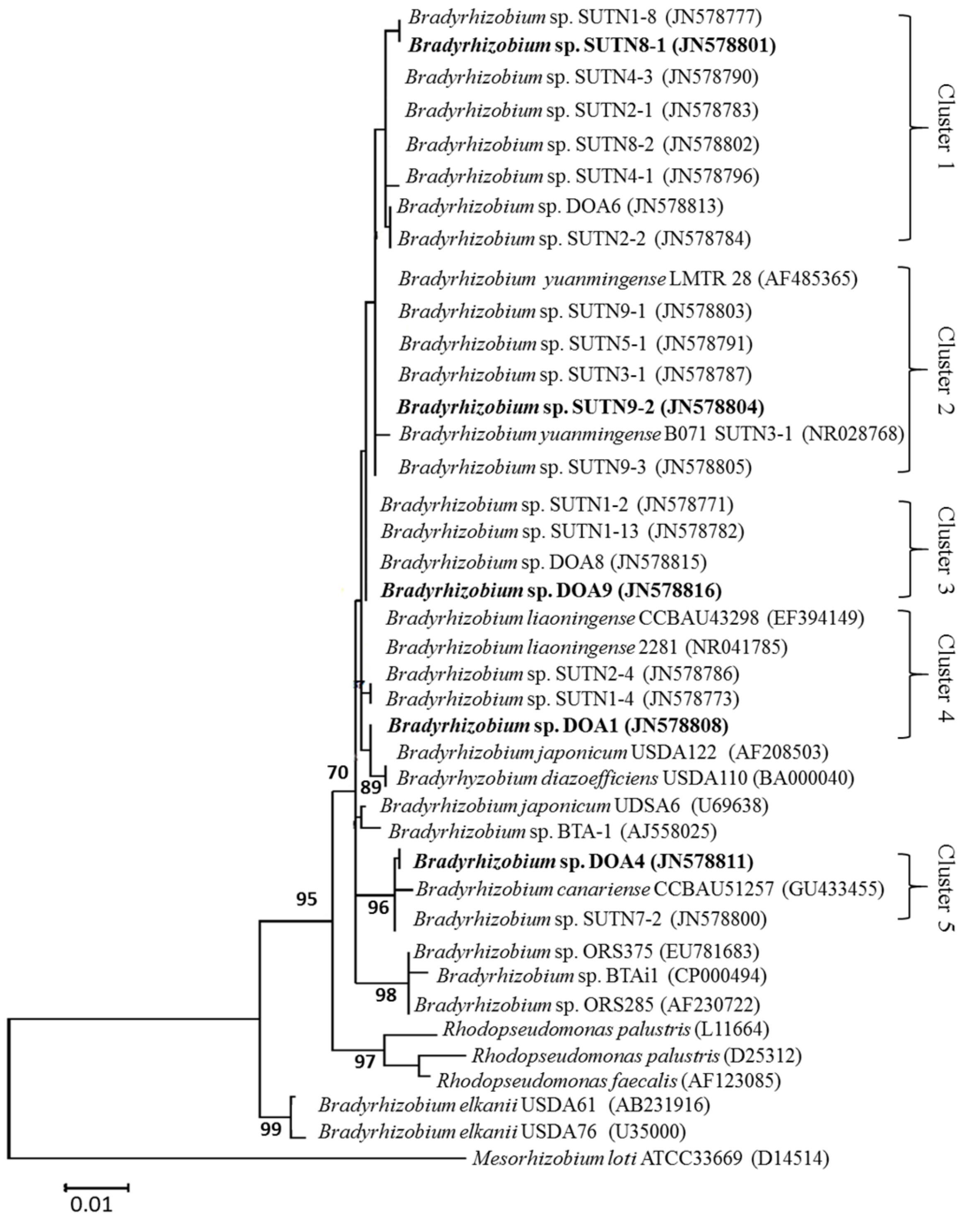


**Fig. S2.** Neighbor-joining trees based on sequences of 16S rRNA genes showing classification of the *Bradyrhizobium* strains isolated from nodules of *A. americana*. Bootstrap values are expressed as percentages of 1,000 replications. The bar represents 1 estimated substitution per 100 nucleotide positions. The evolutionary distances were computed using Kimura two-parameter method and are shown in the units representing the number of base

substitutions per site. This phylogenetic tree was modified from Noisangiam et al., (1). The nucleotide sequences were obtained from NCBI database.

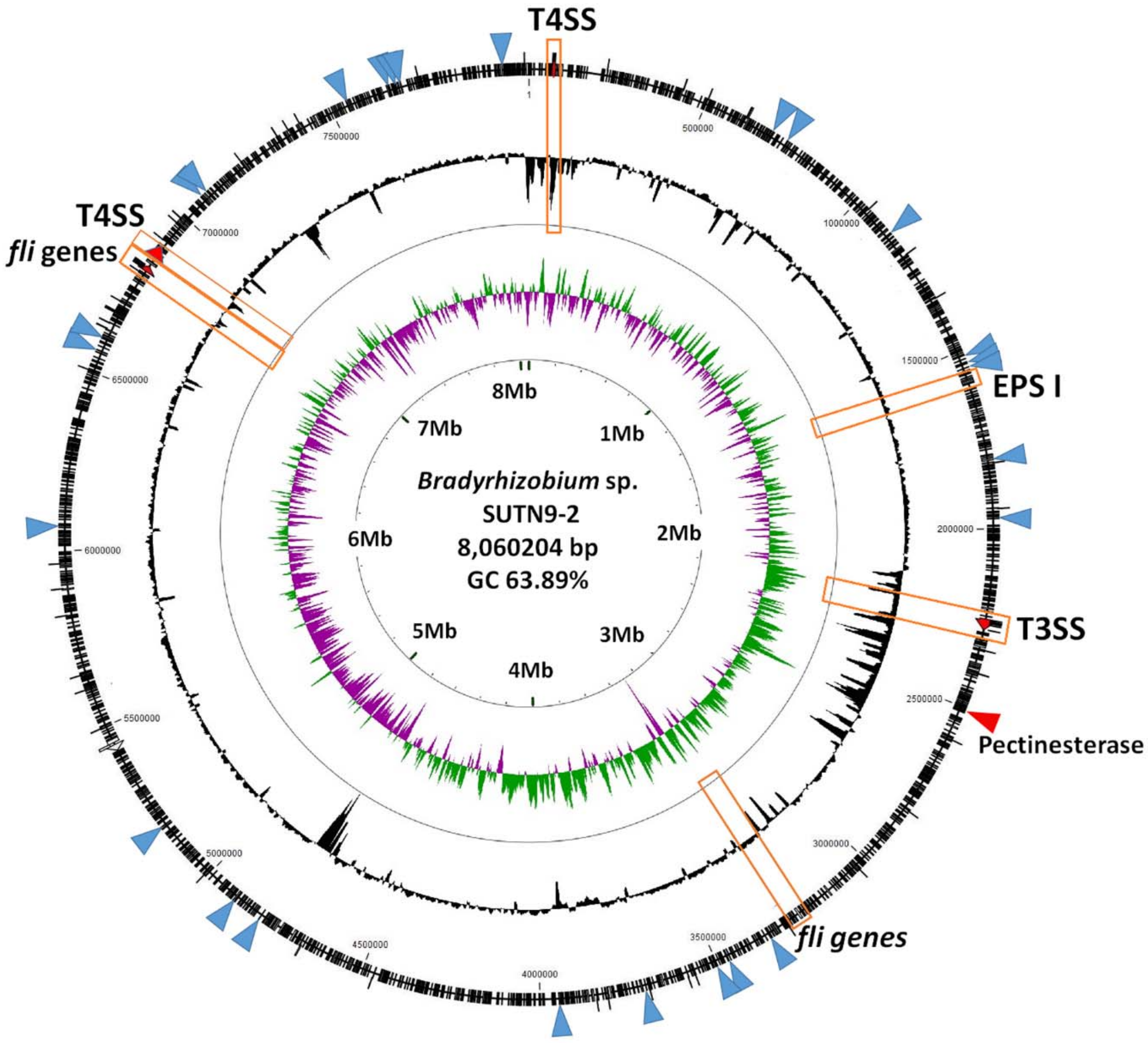


**Fig. S3.** Circular representation of the chromosome of *Bradyrhizobium* sp. SUTN9-2. The scale of outermost circle indicates the location in bp and show the position of the putative protein-encoding genes.  The markings inside the innermost circle represent genome positions (in Mb). The second-innermost and third-innermost circles show the GC skew (green and purple) and the GC content (black), respectively. The positions of the Type III secretion system (T3SS), Type IV secretion system (T4SS), exopolysacharide biosynthesis (EPS I), flagellar biosynthesis (*fli*) cluster genes are shown in orange boxes. The single pectinesterase and 26 glutathione S-transferase (GST) genes are indicated by red and blue triangle on outside the outermost circle, respectively.

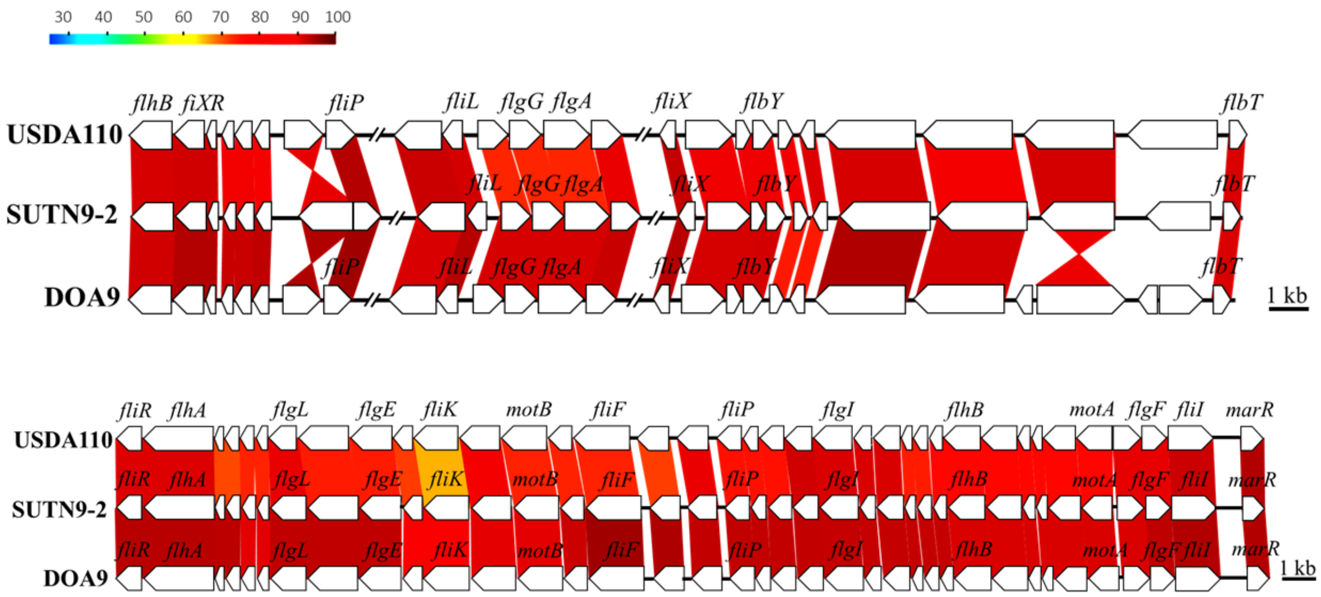


**Fig. S4.** The comparison of the flagella gene cluster including *fliP* between strains USDA110, SUTN9-2 and DOA9. Double slash marks represent DNA regions that are not shown. Colored strips represent the conserved gene regions between the compared strains, and the color indicates the percentage similarity.

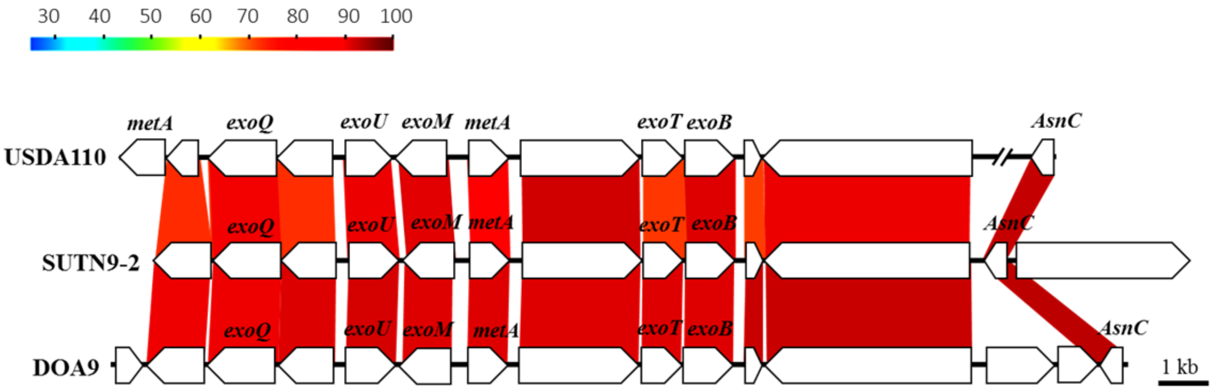


**Fig. S5.** The comparison of the gene cluster for exoporysacharide production including *exoB* between strains USDA110, SUTN9-2 and DOA9. Double slash marks represent DNA regions that are not shown. Colored strips represent the conserved gene regions between the compared strains, and the color indicates the percentage similarity.

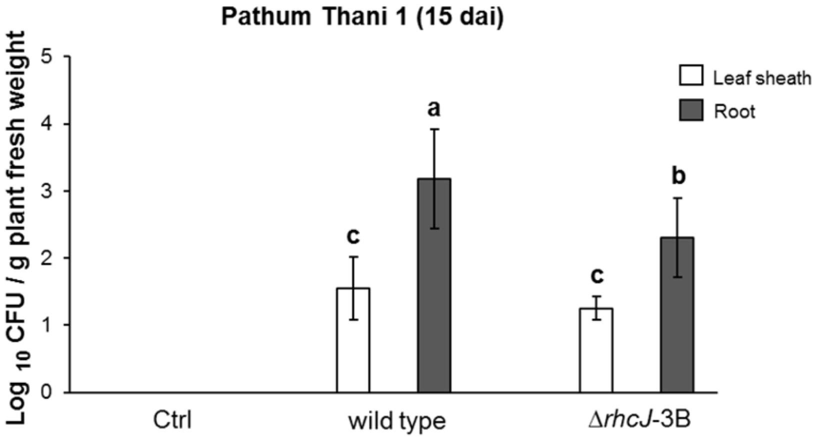


**Fig. S6.** Enumuration of bradyrhizobia in leaf sheath and root of rice tissues. The populations of endophytic bradyrhizobia were examined in the tissues of *O. sativa* L. ssp. *indica* cv. Pathum Thani 1 at 15 day after inoculation (dai). Significance at $P < 0.05$ is indicated by mean standard deviation bars (n = 3).

**Reference**

1) Noisangiam, R., K. Teamtisong, P. Tittabutr, N. Boonkerd, U. Toshiki, K. Minamisawa, and N. Teaumroong. 2012. Genetic diversity, symbiotic evolution, and proposed infection process of *Bradyrhizobium* strains isolated from root nodules of *Aeschynomene americana* L. in Thailand. Appl. Environ. Microbiol. 78:6236-6250.
